# Determinants of linear growth from infancy to school-aged years: a population-based follow-up study in urban Amazonian children

**DOI:** 10.1186/1471-2458-12-265

**Published:** 2012-06-11

**Authors:** Barbara H Lourenço, Eduardo Villamor, Rosângela A Augusto, Marly A Cardoso

**Affiliations:** 1 Public Health Nutrition Program, School of Public Health, University of São Paulo, Avenida Dr. Arnaldo 715, São Paulo, SP, 01246-904, Brazil; 2 Department of Epidemiology, University of Michigan School of Public Health, 1415 Washington Heights, Ann Arbor, MI, 48109-2029, USA; 3 Department of Nutrition, School of Public Health, University of São Paulo, Avenida Dr. Arnaldo 715, São Paulo, SP, 01246-904, Brazil

**Keywords:** Children, Linear growth, Height-for-age Z score, School-aged years, Determinants

## Abstract

**Background:**

Although linear growth during childhood may be affected by early-life exposures, few studies have examined whether the effects of these exposures linger on during school age, particularly in low- and middle-income countries.

**Methods:**

We conducted a population-based longitudinal study of 256 children living in the Brazilian Amazon, aged 0.1 y to 5.5 y in 2003. Data regarding socioeconomic and maternal characteristics, infant feeding practices, morbidities, and birth weight and length were collected at baseline of the study (2003). Child body length/height was measured at baseline and at follow-up visits (in 2007 and 2009). Restricted cubic splines were used to construct average height-for-age Z score (HAZ) growth curves, yielding estimated HAZ differences among exposure categories at ages 0.5 y, 1 y, 2 y, 5 y, 7 y, and 10 y.

**Results:**

At baseline, median age was 2.6 y (interquartile range, 1.4 y–3.8 y), and mean HAZ was −0.53 (standard deviation, 1.15); 10.2% of children were stunted. In multivariable analysis, children in households above the household wealth index median were 0.30 Z taller at age 5 y (*P* = 0.017), and children whose families owned land were 0.34 Z taller by age 10 y (*P* = 0.023), when compared with poorer children. Mothers in the highest tertile for height had children whose HAZ were significantly higher compared with those of children from mothers in the lowest height tertile at all ages. Birth weight and length were positively related to linear growth throughout childhood; by age 10 y, children weighing >3500 g at birth were 0.31 Z taller than those weighing 2501 g to 3500 g (*P* = 0.022) at birth, and children measuring ≥51 cm at birth were 0.51 Z taller than those measuring ≤48 cm (*P* = 0.005).

**Conclusions:**

Results suggest socioeconomic background is a potentially modifiable predictor of linear growth during the school-aged years. Maternal height and child’s anthropometric characteristics at birth are positively associated with HAZ up until child age 10 y.

## Background

Linear growth during childhood may be influenced by the cumulative effects of many environmental exposures, including nutritional, psychosocial, and infectious factors [1]. It is estimated that 167 million children aged <5 y in developing countries (29.2%) were growth-stunted in 2010 [2]. The individual and societal consequences of restricted linear growth are substantial and include increased risks of mortality, infection, neurocognitive delays, and other disabilities [3,4]. In addition, stunted children may become shorter adults with decreased economic productivity and reproductive performance [4,5]. Identifying potentially modifiable causes of linear growth retardation thus remains an important public health priority.

Studies of the determinants of linear growth have focused largely on the period before the age of 5 y [6-8]. However, little is known about the influences of early-life exposures on linear growth during school age, especially in low- and middle-income countries. Understanding these influences would provide critical information as to whether the adverse impact of early exposures on growth lingers on throughout childhood or may be reversed. The aim of this study was to investigate socioeconomic, maternal, and child determinants of linear growth up to age 10 y in a population-based cohort study of children living in the Brazilian Amazon.

## Methods

### Study area and population

This longitudinal study was conducted in Acrelândia, a town located 100 km from Rio Branco, the capital of the state of Acre, in northwest Brazil. Acrelândia is a frontier town inhabited by migrants from southeast and south Brazil who are engaged in commercial agriculture and cattle raising. In 2003, the city had 8697 inhabitants (43% residing in the urban area), covering a territory of 1607.5 km^2^. Child health indicators in Acrelândia are significantly lower than the country average: infant mortality rate was 71/1000 live births, and the Human Development Index was 0.680 according to data from 2000 [9].

In January 2003, we performed a population-based cross-sectional study in the urban area of Acrelândia [10]. All households with children aged <5 y (n = 334) were identified through a census performed by local teams of the Family Health Program of the Brazilian Ministry of Health. All children in these 334 households were invited to participate, and only two households declined participation. Thus, data were collected from 332 households (99.4%) involving a total of 489 children. Structured household interviews and anthropometric evaluations were completed for 468 children (95.7% of those eligible).

As previously reported [11], in December 2007 we conducted a second population-based cross-sectional survey in the same town with all children aged <10 y. The assessment included 250 of the children who had previously been examined in 2003. In December 2009, our group performed another follow-up assessment, which included 193 children evaluated in both 2003 and 2007, plus six other children who participated in 2003 but were not located in 2007. We included in these analyses 256 children who had been evaluated in 2003 and at least once more. These children contributed a total of 705 measurements. The distribution of the number of measurements was as follows: 0 to <6 mo: 21; 6 mo to <12 mo: 27; 12 mo to <2 y: 47; 2 y to <5 y: 162; 5 y to <7 y: 100; 7 y to <10 y: 274; and ≥10 y: 74.

Children included in the analyses were not different from those who were not included in terms of sex, age, length/height, and the socioeconomic, maternal, and child characteristics investigated. Written informed consent for participation was obtained from parents or guardians before enrollment. This study was approved by the ethical review board of the School of Public Health, University of São Paulo, Brazil.

### Field procedures

At baseline of the study (2003), trained fieldworkers performed structured face-to-face interviews with each child’s mother or guardian during household visits. The following data were collected: demographic characteristics (child’s sex and age, race/ethnicity), socioeconomic status (household assets, land ownership, parental educational level) and access to public services (treated water, garbage collection), maternal characteristics (maternal age, smoking during pregnancy), child characteristics at birth (birth weight and length retrieved from child’s health card), infant feeding practices (child’s age at introduction of weaning foods), and morbidity (diarrhea, vomiting, or cough with fever in the 15 days before the home visit).

Trained research assistants obtained anthropometric measurements from the children at a local Family Health Clinic (in 2003 and 2007 surveys) or at the households (in 2009), using standardized procedures and calibrated equipment [12]. The date of birth was recorded directly from birth certificates or child health cards. In 2003, among children aged <24 mo, recumbent length was measured to the nearest millimeter with a locally made infant measuring board. Children aged ≥2 y were measured to the nearest 0.1 cm with a stadiometer (Seca, Hamburg, Germany, in 2003 and 2007; WCS, Curitiba, Brazil, in 2009) affixed to a flat surface on a wall, without a baseboard and perpendicular to the floor. Children were positioned barefoot in the vertical standing position in the middle of the stadiometer, with their head, shoulders, buttocks, and heels against the wall. Mother’s height was measured following similar procedures. Each measurement was repeated, and the mean value was calculated.

### Data analyses

The main outcome of interest was length/height-for-age and sex Z score (HAZ). HAZ was calculated according to the World Health Organization (WHO) Child Growth Standards [13] for children aged 0 to 5 y and the WHO Growth Reference Data [14] for children >5 y. Stunting was defined as HAZ < −2 [15].

Predictors of interest included maternal and household sociodemographic characteristics, child anthropometry at birth, infant feeding practices, and morbidities at baseline. A wealth index based on the presence of 14 home appliances was used to assess socioeconomic status [16]. Principal component analysis was used to define the weight of household assets with the XLSTAT software, version 7.5.2 (Addinsoft, New York, NY). As previously reported [10], after standardizing the weights assigned to each item, scores were added to produce an estimated index of household wealth. In order to account for potential non-linearity of associations, predictors were categorized according to previously used cut-off points in this population. The wealth index was examined in quartiles, tertiles, and as less than or as greater than or equal to the median. As similar results were observed, we opted to present the associations for this variable according to the latter classification. Maternal height and child’s birth length were divided into tertiles, and child’s birth weight was categorized as ≤2500 g, 2501 g to 3500 g, or >3500 g. Age at introduction of cow’s milk, an indicator of infant feeding practices, was classified as <3 mo vs. ≥3 mo.

We first compared the distribution of HAZ by categories of socioeconomic, maternal, and child characteristics at baseline, using tests of trend for ordinal predictors and the mean-comparison t-test for dichotomous predictors. We also examined the prevalence of stunting in relation to baseline characteristics using the Cochrane-Armitage and chi-square tests for ordinal and dichotomous variables, respectively.

Subsequently, the associations of socioeconomic, maternal, and child characteristics with linear growth were assessed by estimating average HAZ-for-age growth curves for each category of the predictors of interest, using mixed-effect models for repeated measurements with restricted cubic splines. Cubic splines represent non-linear terms for age at each assessment that allow the smoothing of the relation between HAZ and age. Piecewise cubic polynomials are smoothly joined at joint points or “knots” [17]. In this case, knots were placed at ages 0.18 y, 0.50 y, 1.50 y, 3 y, and 10 y, as these ages seem important reference points in the curvilinear segments of the WHO Child Growth Standard and Reference Data [13,14]. In the models, the outcome was HAZ, and covariates included the categories of the predictor of interest, linear and spline terms for child age in decimal years, and predictor category*age interaction terms. Random effects for the intercept and the linear term for age (slope) were included to account for the within-person correlation of measurements in the estimation of the variance [18]. Since these methods do not require an equal number of measurements in all children or that measurements be obtained at exactly the same time points on every participant, all available measurements were included in the models. Because the age distribution of children at baseline ranged from 0 to 5 y, we tested for possible birth cohort effects on the construction of the curves by including additional terms for year of birth. These terms were not statistically significant and did not change the magnitude of the associations.

We obtained adjusted mean HAZ-for-age curves using multivariable mixed-effect models based on a hierarchical conceptual framework as proposed by Victora et al [19]. The following levels of determination were considered: (1) socioeconomic characteristics, (2) access to public services, (3) maternal characteristics before pregnancy, (4) maternal characteristics during pregnancy, (5) child characteristics at birth, (6) infant feeding practices, and (7) morbidity indicators. For each level, variables were retained in the model if they were considered conceptually relevant or if there was a clear association with the outcome in the unadjusted analysis, including dose–response patterns for ordinal variables. Statistical significance (*P* < 0.100) was an additional criterion for retaining a variable in the model at each level of determination.

For both unadjusted and adjusted analyses, we estimated HAZ from the growth curves at ages 6 mo, 12 mo, 2 y, 5 y, 7 y, and 10 y, as the predicted values of the spline function, with values of predictor covariates at the reference category. Differences in the estimated values of HAZ and their 95% confidence intervals (CI) were calculated among the categories of each predictor at these ages. All reported *P* values are two-tailed. We used SAS 9.2 (SAS Institute Inc., Cary, NC) for all analyses.

## Results

Median age at recruitment was 2.6 y (interquartile range [IQR], 1.4 y–3.8 y; range, 0.1 y–5.5 y), 52.7% of the children were female, and 88.1% were mulatto. At baseline, mean HAZ was −0.53 (standard deviation [SD], 1.15), and 10.2% of the children were stunted. In 2003, HAZ was positively associated with wealth index, maternal education and height, non-smoking during pregnancy, and child’s birth weight and length. No association with child’s sex was observed (Table 1).

**Table 1:** Mean height-for-age Z score and prevalence of stunting according to baseline characteristics, Acrelândia, Brazil

	**n**^1^	**Mean HAZ (SD)**^2^	***P***^3^	**% stunted**^4^	***P***^5^
**Overall**	**256**	**−0.53 (1.15)**		**10.2**	
Child’s sex			0.376		0.866
Female	132	−0.59 (1.10)		9.8	
Male	124	−0.47 (1.21)		10.5	
Child’s age			<0.001		0.875
0-5 months	21	−0.24 (1.33)		19.0	
6-11 months	27	0.30 (1.04)		0.0	
12-23 months	47	−0.41 (1.17)		8.5	
24-35 months	56	−0.83 (1.04)		12.5	
≥36 months	105	−0.70 (1.09)		10.5	
**Socioeconomic characteristics**					
Wealth index			0.008		0.035
Below median	136	−0.70 (1.16)		14.0	
Above median	118	−0.32 (1.12)		5.9	
Land ownership			0.035		0.136
No	208	−0.60 (1.16)		11.5	
Yes	47	−0.21 (1.09)		4.3	
Mother’s educational level			0.003		0.074
0-4 years	102	−0.68 (1.12)		11.8	
≥5 years	88	−0.19 (1.12)		4.5	
Access to treated water			0.866		0.242
No	82	−0.54 (1.34)		13.4	
Yes	173	−0.52 (1.06)		8.7	
Access to public garbage collection			0.206		0.005
No	53	−0.70 (1.40)		20.8	
Yes	199	−0.47 (1.08)		7.5	
**Maternal characteristics**					
Mother’s age			0.915		0.468
≤20 years	27	−0.45 (1.28)		18.5	
21-30 years	143	−0.51 (1.14)		9.1	
>30 years	55	−0.50 (1.28)		10.9	
Mother’s height			<0.001		0.008
1^st^ tertile: ≤154.0 cm	77	−0.95 (1.16)		18.2	
2^nd^ tertile: 154.1-159.4 cm	77	−0.55 (1.02)		7.8	
3^rd^ tertile: ≥159.5 cm	78	−0.22 (1.10)		5.1	
Smoking during pregnancy			0.016		0.193
No	176	−0.43 (1.18)		8.5	
Yes	55	−0.85 (1.00)		14.5	
**Child characteristics**					
Child’s birth weight			<0.001		0.013
≤2500 g	18	−1.45 (0.73)		16.7	
2501-3500 g	156	−0.66 (1.11)		12.2	
>3500 g	76	−0.01 (1.08)		2.6	
Child’s birth length			<0.001		0.003
1^st^ tertile: ≤48 cm	77	−0.96 (1.06)		14.3	
2^nd^ tertile: 49–50 cm	73	−0.17 (1.00)		4.1	
3^rd^ tertile: ≥51 cm	39	0.15 (1.25)		0.0	
Age at cow’s milk introduction			0.689		0.867
<3 months	77	−0.60 (1.15)		10.4	
≥3 months	165	−0.53 (1.14)		9.7	
Morbidities 15 days before baseline visit:					
Diarrhea			0.892		0.985
No	195	−0.53 (1.19)		10.3	
Yes	59	−0.56 (1.04)		10.2	
Vomiting			0.291		0.910
No	233	−0.51 (1.16)		10.3	
Yes	21	−0.79 (0.99)		9.5	
Cough with fever			0.206		0.951
No	222	−0.50 (1.17)		10.4	
Yes	30	−0.79 (1.03)		10.0	

1. Totals may differ from 256 due to missing values.

2. HAZ: height-for-age Z score, calculated according to the WHO growth references [13,14].

3. Test for linear trend for ordinal predictors; mean-comparison t-test for dichotomous predictors.

4. Stunting: HAZ < −2, classified according to the WHO reference [15].

5. Cochrane-Armitage test for trend for ordinal predictors; chi-square test for dichotomous predictors.

**Figure 1:**
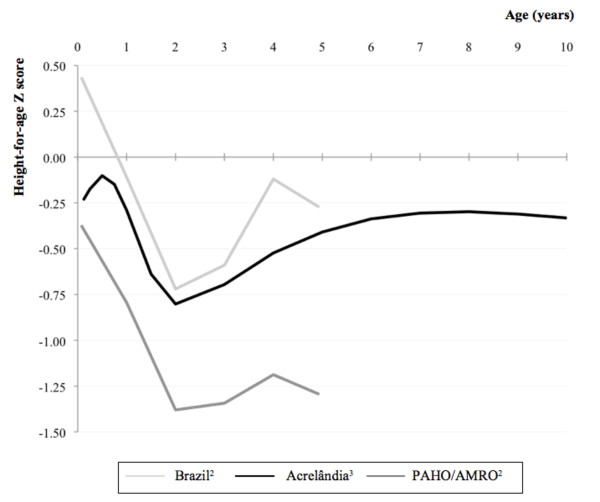
**Mean height-for-age by age for Acrelândia in comparison to mean height-for-age by age for Brazil and Latin America and the Caribbean (PAHO/AMRO) region**^1^**.** 1. Height-for-age Z scores in all curves were calculated according to the WHO growth references [13,14]. Height-for-age Z score curves are compared to the WHO growth references, whose median height-for-age values by age form the reference horizontal line at 0.00. 2. Curves estimated based on information from the WHO Global Database on Child Growth and Malnutrition [20]. 3. Curve estimated based on restricted cubic spline regression models.

Median follow-up time was 6.9 y (IQR, 6.8 y–6.9 y), during which time a median of three height measurements was collected for each child (IQR, 3–3; 63 children had two and 193 children had three measurements). We estimated an average HAZ-for-age curve for the study population (Figure 1) from age 0 to 10 years using restricted cubic splines. The curve’s shape was comparable with curves for Brazil and for the Latin America and the Caribbean region (PAHO/AMRO) estimated from the WHO Database on Child Growth and Malnutrition, which is available for children aged <5 y [20]. Brazilian and PAHO/AMRO estimates are derived from nationally representative demographic and health cross-sectional surveys conducted from 1999 to 2006. For all ages, HAZ values of Acrelândia children were slightly lower than those from the Brazilian national average, but above the PAHO/AMRO regional mean.

In unadjusted analyses (Table 2), socioeconomic status, land ownership, and maternal education were positively associated with mean HAZ values at ages 2 y, 5 y, 7 y, and 10 y. Monotonically increasing children’s mean HAZ were observed across categories of mother’s height, child’s birth weight, and child’s birth length. Linear growth patterns did not differ by sex.

**Table 2:** Height-for-age Z scores by socioeconomic, maternal and child characteristics, Acrelândia, Brazil, unadjusted analysis

		**Mean HAZ (SE) according to age**^2,3^					
	**n**^1^	**6 mo**	**12 mo**	**2 y**	**5 y**	**7 y**	**10 y**
Child’s sex	256						
Female		−0.25 (0.18)	−0.31 (0.19)	−0.90 (0.13)	−0.41 (0.08)	−0.32 (0.07)	−0.42 (0.08)
Male		0.09 (0.27)	−0.25 (0.17)	−0.74 (0.13)	−0.40 (0.09)	−0.29 (0.09)	−0.26 (0.09)
Difference [95% CI]		0.34 [−0.29, 0.97]	−0.06 [−0.44, 0.56]	0.16 [−0.20, 0.52]	−0.01 [−0.23, 0.25]	0.03 [−0.20, 0.26]	0.16 [−0.08, 0.40]
**Socioeconomic characteristics**							
Wealth index	254						
Below median		−0.33 (0.20)	−0.40 (0.17)	−0.99 (0.12)	−0.57 (0.08)	−0.44 (0.08)	−0.43 (0.08)
Above median		0.20 (0.24)	−0.16 (0.19)	−0.61 (0.14)	−0.21 (0.09)	−0.13 (0.08)	−0.20 (0.09)
Difference		0.53 [−0.08, 1.15]	0.24 [−0.26, 0.74]	0.38 [0.02, 0.74]	0.36 [0.12, 0.60]	0.31 [0.09, 0.54]	0.23 [−0.01, 0.47]
Land ownership	255						
No		−0.19 (0.16)	−0.38 (0.14)	−0.88 (0.10)	−0.48 (0.07)	−0.38 (0.06)	−0.41 (0.07)
Yes		0.47 (1.10)	0.08 (0.38)	−0.52 (0.20)	−0.08 (0.13)	0.04 (0.12)	0.00 (0.13)
Difference		0.66 [−1.52, 2.85]	0.46 [−0.32, 1.25]	0.36 [−0.09, 0.81]	0.40 [0.11, 0.69]	0.42 [0.14, 0.69]	0.41 [0.12, 0.70]
Mother’s educational level	190						
0-4 years		−0.01 (0.18)	−0.23 (0.16)	−1.02 (0.14)	−0.56 (0.09)	−0.42 (0.09)	−0.41 (0.10)
≥5 years		0.08 (0.19)	−0.14 (0.21)	−0.41 (0.17)	−0.12 (0.10)	−0.08 (0.10)	−0.17 (0.10)
Difference		0.09 [−0.43, 0.61]	0.09 [−0.42, 0.61]	0.61 [0.19, 1.03]	0.44 [0.18, 0.70]	0.33 [0.08, 0.59]	0.24 [−0.04, 0.51]
Access to treated water	255						
No		0.25 (0.24)	−0.30 (0.21)	−0.85 (0.19)	−0.58 (0.11)	−0.48 (0.10)	−0.43 (0.11)
Yes		−0.25 (0.18)	−0.35 (0.16)	−0.80 (0.10)	−0.32 (0.07)	−0.22 (0.07)	−0.28 (0.07)
Difference		−0.50 [−1.09, 0.09]	−0.05 [−0.57, 0.46]	0.05 [−0.37, 0.46]	0.26 [−0.01, 0.52]	0.26 [0.01, 0.50]	0.14 [−0.12, 0.40]
Access public garbage collection	252						
No		−0.52 (0.39)	−0.45 (0.27)	−0.78 (0.27)	−0.70 (0.16)	−0.60 (0.15)	−0.44 (0.14)
Yes		−0.02 (0.17)	−0.28 (0.15)	−0.80 (0.09)	−0.33 (0.07)	−0.23 (0.06)	−0.31 (0.07)
Difference		0.50 [−0.32, 1.33]	0.17 [−0.44, 0.77]	−0.02 [−0.58, 0.54]	0.37 [0.04, 0.70]	0.37 [0.05, 0.68]	0.12 [−0.18, 0.43]
**Maternal characteristics**							
Mother’s height	232						
a. 1^st^ tertile: ≤154.0 cm		−0.67 (0.26)	−0.59 (0.23)	−1.17 (0.15)	−0.78 (0.12)	−0.64 (0.11)	−0.61 (0.11)
b. 2^nd^ tertile: 154.1-159.4 cm		−0.20 (0.35)	−0.38 (0.23)	−0.81 (0.17)	−0.36 (0.09)	−0.29 (0.09)	−0.42 (0.11)
c. 3^rd^ tertile: ≥159.5 cm		0.33 (0.25)	0.06 (0.24)	−0.58 (0.16)	−0.19 (0.11)	−0.06 (0.10)	−0.05 (0.09)
Difference (b-a)		0.47 [−0.37, 1.32]	0.21 [−0.41, 0.84]	0.35 [−0.10, 0.80]	0.41 [0.12, 0.71]	0.35 [0.07, 0.63]	0.18 [−0.13, 0.49]
Difference (c-a)		1.00 [0.30, 1.70]	0.65 [0.01, 1.29]	0.58 [0.15, 1.01]	0.59 [0.28, 0.90]	0.58 [0.28, 0.88]	0.56 [0.27, 0.84]
*P* for trend		0.458	0.412	0.323	0.314	0.313	0.299
Smoking during pregnancy	231						
No		0.06 (0.19)	−0.17 (0.15)	−0.70 (0.11)	−0.35 (0.08)	−0.26 (0.07)	−0.32 (0.07)
Yes		−0.52 (0.26)	−0.65 (0.23)	−1.23 (0.16)	−0.64 (0.12)	−0.49 (0.12)	−0.51 (0.13)
Difference		−0.58 [−1.21, 0.05]	−0.48 [−1.03, 0.05]	−0.53 [−0.91, −0.15]	−0.29 [−0.57, −0.02]	−0.22 [−0.49, 0.05]	−0.19 [−0.49, 0.10]
**Child characteristics**							
Child’s birth weight	250						
a. ≤2500 g		−1.23 (0.34)	−1.14 (0.35)	−1.89 (0.25)	−0.97 (0.12)	−0.74 (0.13)	−0.81 (0.16)
b. 2501-3500 g		−0.27 (0.19)	−0.47 (0.16)	−0.94 (0.12)	−0.53 (0.08)	−0.42 (0.08)	−0.43 (0.08)
c. >3500 g		0.77 (0.17)	0.25 (0.19)	−0.30 (0.14)	0.02 (0.10)	0.08 (0.10)	−0.01 (0.11)
Difference (a-b)		−0.96 [−1.72, −0.21]	−0.67 [−1.42, 0.08]	−0.95 [−1.50, −0.40]	−0.44 [−0.72, −0.16]	−0.32 [−0.62, −0.03]	−0.39 [−0.74, −0.03]
Difference (c-b)		1.04 [0.54, 1.54]	0.72 [0.23, 1.21]	0.64 [0.27, 1.01]	0.55 [0.30, 0.80]	0.50 [0.26, 0.73]	0.42 [0.16, 0.67]
* P* for trend		0.163	0.146	0.108	0.061	0.050	0.043
Child’s birth length	189						
a. 1^st^ tertile: ≤48 cm		−0.60 (0.27)	−0.69 (0.23)	−1.46 (0.15)	−0.63 (0.11)	−0.48 (0.10)	−0.67 (0.10)
b. 2^nd^ tertile: 49–50 cm		0.44 (0.21)	−0.12 (0.19)	−0.52 (0.13)	−0.23 (0.09)	−0.15 (0.09)	−0.16 (0.10)
c. 3^rd^ tertile: ≥51 cm		1.20 (0.28)	0.36 (0.29)	−0.15 (0.24)	0.12 (0.18)	0.18 (0.16)	0.11 (0.16)
Difference (a-b)		−1.04 [−1.71, 0.37]	−0.57 [−1.15, 0.00]	−0.94 [−1.33, −0.54]	−0.40 [−0.68, −0.13]	−0.33 [−0.60, −0.07]	−0.51 [−0.80, −0.22]
Difference (c-b)		0.76 [0.06, 1.45]	0.47 [−0.21, 1.15]	0.37 [−0.17, 0.91]	0.35 [−0.05, 0.75]	0.32 [−0.04, 0.69]	0.26 [−0.11, 0.64]
* P* for trend		0.051	0.053	0.053	0.019	0.016	0.020
Age at cow’s milk introduction	242						
<3 months		−0.05 (0.27)	−0.44 (0.24)	−1.00 (0.15)	−0.48 (0.09)	−0.36 (0.09)	−0.44 (0.10)
≥3 months		−0.12 (0.21)	−0.22 (0.16)	−0.79 (0.11)	−0.42 (0.08)	−0.31 (0.08)	−0.32 (0.08)
Difference		0.07 [−0.60, 0.74]	−0.22 [−0.79, 0.34]	−0.21 [−0.58, 0.15]	−0.06 [−0.30, 0.18]	−0.05 [−0.28, 0.18]	−0.12 [−0.36, 0.13]
Morbidities 15 days before baseline visit:							
Diarrhea	254						
No		−0.01 (0.19)	−0.21 (0.16)	−0.83 (0.11)	−0.41 (0.07)	−0.29 (0.07)	−0.30 (0.07)
Yes		−0.26 (0.29)	−0.42 (0.22)	−0.78 (0.16)	−0.41 (0.10)	−0.37 (0.09)	−0.52 (0.11)
Difference		−0.24 [−0.92, 0.43]	−0.21 [−0.74, 0.32]	0.05 [−0.33, 0.43]	0.00 [−0.23, 0.24]	−0.08 [−0.30, 0.15]	−0.22 [−0.48, 0.04]
Vomiting	254						
No		−0.03 (0.17)	−0.21 (0.13)	−0.79 (0.10)	−0.43 (0.07)	−0.32 (0.06)	−0.33 (0.06)
Yes		−0.58 (0.30)	−0.81 (0.33)	−1.31 (0.29)	−0.32 (0.15)	−0.19 (0.14)	−0.48 (0.16)
Difference		−0.55 [−1.23, 0.13]	−0.61 [−1.30, 0.09]	−0.52 [−1.13, 0.08]	0.10 [−0.22, 0.43]	0.13 [−0.17, 0.44]	−0.15 [−0.48, 0.19]
Cough with fever	252						
No		−0.07 (0.17)	−0.24 (0.14)	−0.76 (0.10)	−0.40 (0.07)	−0.29 (0.06)	−0.30 (0.07)
Yes		−0.21 (0.38)	−0.53 (0.31)	−1.35 (0.21)	−0.57 (0.12)	−0.44 (0.13)	−0.65 (0.14)
Difference		−0.14 [−0.97, 0.68]	−0.28 [−0.96, 0.39]	−0.59 [−1.04, −0.13]	−0.17 [−0.45, 0.11]	−0.15 [−0.43, 0.14]	−0.35 [−0.65, −0.05]

1. Totals may differ from 256 due to missing values.

2. HAZ: height-for-age Z score, calculated according to the WHO growth references [13,14].

3. Unadjusted mean HAZ values and standard errors were estimated from restricted cubic spline regression models. The age distribution of available measurements used in these models was: 0 to <6 mo: 21; 6 mo to <12 mo: 27; 12 mo to <2 y: 47; 2 y to <5 y: 162; 5 y to <7 y: 100; 7 y to <10 y: 274; and ≥10 y: 74.

After multivariable adjustment, wealth and land ownership remained significantly related to HAZ (Table 3). At ages 5 y and 7 y, children from households above the wealth index median had a 0.30 and 0.25 higher HAZ (*P* = 0.017 and *P* = 0.034, respectively) compared with children from households below the median. Land ownership was positively related to HAZ during the school-aged years; by age 10 y, children whose families owned land were 0.34 Z taller (*P* = 0.023) than children from landless families. Inclusion of mother’s educational level did not appreciably change the estimates, and, after adjusting for socioeconomic variables, access to public services was not found to be related to HAZ.

**Table 3:** Multivariable models for height-for-age Z scores by socioeconomic, maternal and child characteristics, Acrelândia, Brazil

	**Adjusted mean HAZ (SE) according to age**^1,2^					
	**6 mo**	**12 mo**	**2 y**	**5 y**	**7 y**	**10 y**
**MODEL 1** (n = 231)^3^						
Wealth index						
Below median	−0.37 (0.20)	−0.43 (0.17)	−1.01 (0.12)	−0.60 (0.09)	−0.47 (0.08)	−0.47 (0.08)
Above median	0.07 (0.25)	−0.29 (0.21)	−0.68 (0.16)	−0.30 (0.10)	−0.23 (0.09)	−0.30 (0.10)
Difference [95% CI]	0.44 [−0.18, 1.06]	0.14 [−0.39, 0.67]	0.33 [−0.06, 0.72]	0.30 [0.06, 0.54]	0.25 [0.02, 0.48]	0.17 [−0.07, 0.40]
Land ownership						
No	−0.37 (0.20)	−0.43 (0.17)	−1.01 (0.12)	−0.60 (0.09)	−0.47 (0.08)	−0.47 (0.08)
Yes	0.16 (1.12)	0.00 (0.46)	−0.77 (0.27)	−0.29 (0.16)	−0.14 (0.15)	−0.13 (0.15)
Difference	0.53 [−1.68, 2.73]	0.43 [−0.41, 1.28]	0.24 [−0.24, 0.73]	0.31 [0.01, 0.61]	0.33 [0.06, 0.61]	0.34 [0.05, 0.63]
**MODEL 2** (n = 231)^4^						
Mother’s height						
a. 1^st^ tertile: ≤154.0 cm	−1.12 (0.33)	−0.73 (0.26)	−1.33 (0.17)	−0.95 (0.13)	−0.80 (0.13)	−0.72 (0.12)
b. 2^nd^ tertile: 154.1-159.4 cm	−0.48 (0.33)	−0.45 (0.24)	−0.95 (0.17)	−0.52 (0.11)	−0.43 (0.10)	−0.52 (0.12)
c. 3^rd^ tertile: ≥159.5 cm	−0.02 (0.30)	−0.12 (0.30)	−0.73 (0.19)	−0.38 (0.12)	−0.25 (0.12)	−0.18 (0.11)
Difference (b-a)	0.64 [−0.16, 1.43]	0.28 [−0.35, 0.91]	0.37 [−0.07, 0.82]	0.43 [0.14, 0.72]	0.37 [0.09, 0.64]	0.20 [−0.10, 0.51]
Difference (c-a)	1.10 [0.35, 1.84]	0.61 [−0.06, 1.28]	0.60 [0.17, 1.03]	0.57 [0.27, 0.87]	0.55 [0.27, 0.84]	0.54 [0.26, 0.81]
*P* for trend	0.252	0.222	0.172	0.157	0.153	0.147
**MODEL 3** (n = 226)^5^						
Child’s birth weight						
a. ≤2500 g	−1.78 (0.50)	−1.43 (0.47)	−2.13 (0.30)	−1.22 (0.13)	−0.96 (0.14)	−0.94 (0.16)
b. 2501-3500 g	−1.23 (0.31)	−0.76 (0.26)	−1.35 (0.18)	−1.03 (0.13)	−0.88 (0.13)	−0.78 (0.14)
c. >3500 g	−0.03 (0.33)	−0.25 (0.31)	−0.84 (0.22)	−0.50 (0.17)	−0.42 (0.16)	−0.47 (0.16)
Difference (a-b)	−0.55 [−1.53, 0.43]	−0.67 [−1.64, 0.30]	−0.78 [−1.39, −0.16]	−0.19 [−0.49, 0.10]	−0.08 [−0.39, 0.22]	−0.16 [−0.51, 0.19]
Difference (c-b)	1.20 [0.62, 1.78]	0.51 [−0.03, 1.05]	0.51 [0.14, 0.88]	0.53 [0.26, 0.78]	0.46 [0.21, 0.70]	0.31 [0.05, 0.57]
* P* for trend	0.279	0.262	0.221	0.180	0.162	0.141
**MODEL 4** (n = 168)^6^						
Child’s birth length						
a. 1^st^ tertile: ≤48 cm	−1.10 (0.47)	−0.98 (0.33)	−1.76 (0.20)	−0.97 (0.16)	−0.79 (0.16)	−0.88 (0.15)
b. 2^nd^ tertile: 49–50 cm	0.18 (0.48)	−0.93 (0.30)	−1.00 (0.22)	−0.76 (0.17)	−0.66 (0.15)	−0.56 (0.17)
c. 3^rd^ tertile: ≥51 cm	1.07 (0.65)	−0.42 (0.36)	−0.72 (0.28)	−0.42 (0.21)	−0.35 (0.19)	−0.37 (0.18)
Difference (a-b)	−1.28 [−1.91, −0.64]	−0.05 [−0.59, 0.50]	−0.76 [−1.18, -0.34]	−0.21 [−0.52, 0.09]	−0.13 [−0.41, 0.16]	−0.32 [−0.63, −0.01]
Difference (c-b)	0.89 [−0.03, 1.80]	0.51 [−0.24, 1.26]	0.28 [−0.22, 0.79]	0.34 [−0.03, 0.70]	0.31 [−0.03, 0.64]	0.19 [−0.16, 0.54]
*P* for trend	<0.001	<0.001	<0.001	<0.001	<0.001	<0.001
**MODEL 5** (n = 213)^7^						
Age at cow’s milk introduction						
<3 months	−1.62 (0.37)	−1.20 (0.35)	−1.52 (0.19)	−1.11 (0.15)	−0.98 (0.14)	−0.93 (0.16)
≥3 months	−1.75 (0.30)	−0.62 (0.29)	−1.35 (0.21)	−1.08 (0.15)	−0.94 (0.14)	−0.84 (0.15)
Difference	0.13 [−0.51, 0.79]	−0.58 [−1.22, 0.06]	−0.17 [−0.53, 0.20]	−0.03 [−0.26, 0.20]	−0.04 [−0.26, 0.18]	−0.09 [−0.34, 0.15]
**MODEL 6** (n = 210)^8^						
Diarrhea 15 days before baseline visit						
No	−1.80 (0.36)	−0.46 (0.28)	−1.32 (0.22)	−1.12 (0.16)	−0.96 (0.15)	−0.80 (0.16)
Yes	−2.26 (0.49)	−0.47 (0.37)	−1.18 (0.27)	−1.13 (0.17)	−1.07 (0.16)	−1.04 (0.17)
Difference	−0.46 [−1.27, 0.34]	−0.01 [−0.63, 0.61]	0.14 [−0.28, 0.56]	−0.01 [−0.25, 0.23]	−0.11 [−0.35, 0.13]	−0.24 [−0.50, 0.02]

1. HAZ: height-for-age Z score, calculated according to the WHO growth references [13,14].

2. Adjusted mean HAZ values and standard errors were estimated from restricted cubic spline regression models. The age distribution of available measurements used in these models was: 0 to <6 mo: 21; 6 mo to <12 mo: 27; 12 mo to <2 y: 47; 2 y to <5 y: 162; 5 y to <7 y: 100; 7 y to <10 y: 274; and ≥10 y: 74.

3. Co-variates in the model: wealth index and land ownership.

4. Co-variates in the model: model 1 plus mother’s height.

5. Co-variates in the model: model 2 plus child’s birth weight.

6. Co-variates in the model: model 2 plus child’s birth length.

7. Co-variates in the model: model 3 plus age at cow’s milk introduction.

8. Co-variates in the model: model 5 plus morbidities in the past 15 days.

Maternal height was related to HAZ at all ages. Mothers in the highest tertile of height had children whose HAZ was significantly higher compared with those of children whose mothers were in the lowest height tertile after adjusting for socioeconomic status. Smoking during pregnancy was not related to growth in height after adjusting for other covariates.

Birth weight and birth length were positively and significantly related to linear growth throughout childhood. By age 10 y, children weighing >3500 g at birth were 0.31 Z taller (*P* = 0.022) than those who weighed 2501 g to 3500 g. However, low-birth-weight babies were not significantly shorter than normal-weight babies after age 2 y. Shorter babies remained short during the school-aged years; the HAZ difference between extreme tertiles of birth length was 0.51 by age 10 y (*P* = 0.005). In a subsample comprising of 168 children with both birth weight and birth length information, the association between birth weight and linear growth was attenuated after further adjustment for birth length. By age 10 y, the HAZ difference between children weighing >3500 g and children weighing 2501 g to 3500 g at birth decreased to 0.19 (*P* = 0.315).

Early introduction of cow’s milk and the occurrence of diarrhea were related to HAZ at 12 mo and 10 y, respectively, but these associations were not statistically significant (*P* = 0.078 and *P* = 0.069, respectively).

## Discussion

In this population-based cohort study of children from the Brazilian Amazon, socioeconomic background was positively related to linear growth during the school-aged years, whereas maternal height and child birth weight and length were associated with height up until age 10 y.

Previous prospective studies in developed and developing countries have shown associations between socioeconomic variables and attained height. The 1958 British birth cohort found that manual social class, family size, and household crowding were inversely related to considerable differences in height at ages 7 y, 11 y, 16 y, and 33 y [21]. Although in past decades these characteristics affected linear growth throughout childhood, results from a more recent generation of British children have shown that socioeconomic disparities now have a major impact on birth length – an indication that, in a high-income setting, socioeconomic position expresses its effects on height mostly through mechanisms before birth rather than during childhood [22].

Among low- to middle-income settings, nationally representative studies performed in India and Thailand have concluded that linear growth retardation is disproportionately concentrated among children from poor households [23,24], but few studies have been conducted in cohorts followed for several years. Our findings indicate that the socioeconomic background is an important predictor of linear growth in this population of pre-school and school-aged children from the Brazilian Amazon. Interventions to ameliorate poverty could have positive effects on linear growth. A conditional cash transfer program in Mexico was found to enhance linear growth among infants by approximately 0.40 Z after 2 year’s implementation [25]. It is unknown whether this effect can be sustained through school age. Four Brazilian cross-sectional national household surveys performed over a 33-year period showed a steep decline in the overall prevalence of stunting among children aged <5 y due to economic growth coupled with equity-oriented public policies and improvements in the population’s purchasing power, maternal education, sanitation, and access to health care [26]. These surveys, however, did not include older children, nor were they representative of Amazonian populations.

Our results are consistent with the literature regarding the constant and positive association of maternal height with child’s linear growth. An analysis of 109 cross-sectional demographic and health surveys in 54 low- to middle-income countries confirmed that maternal stature is inversely associated with the likelihood of stunting of offspring up until age 5 y [27]. Among these Brazilian Amazon children, and as reported from Great Britain [21] and Pelotas [8] birth cohorts, maternal nutrition may represent the combined effects of genetics and early-life environmental factors, reflecting the intergenerational transfer of both socioeconomic conditions and biologic mechanisms that have consequences for child health. For example, shorter mothers might provide an inadequate supply of nutrients to their fetuses and have narrower pelvises, thereby increasing risk for deliveries with complications [28].

Concerning perinatal exposures, child’s birth weight and length were strong and positive determinants of HAZ throughout school age, in agreement with previous studies [6,8,29]. Although birth weight is in some ways conditioned to maternal height [21], it is noteworthy that the association of birth weight with HAZ was virtually unchanged after controlling for socioeconomic and maternal characteristics, suggesting that the influence of birth weight is independent of maternal stature. Consistent with this notion, a cohort of Belgian monozygotic twin girls (allowing control for genetic and maternal factors) found that the twin who was at least 5% heavier at birth was also taller as an adult [30].

In our study, early introduction of cow’s milk was not significantly associated with HAZ. Only a few previous longitudinal investigations have reported long-term associations between infant feeding practices and anthropometric outcomes [8,21,31]. Evidence linking untimely introduction of cow’s milk with diseases such as type 1 diabetes [32] suggests that complementary feeding should provide appropriate foods in addition to breast milk at around age 6 mo [33,34] to ensure satisfactory nutritional status during infancy.

The present findings should be considered in light of the limitations and strengths of our study. Although this was a population-based study, sample size was relatively small. Because of the high mobility of Acrelândia’s residents, mostly driven by job offers, the follow-up rate was 55% (256 of 468 children who participated at baseline). However, children included in the analyses were not statistically different from those who were not included with respect to sex, age, length/height, and the socioeconomic, maternal, and child characteristics at baseline. Another limitation is that we lacked information on child’s father. Birth weight and length were obtained from child health cards rather than through direct measurement by the research team. Nonetheless, there is evidence that these birth weight records have high validity in Brazil [35]. There are several strengths to the study, including its longitudinal design, the extended follow-up period, the large number of determinants examined, and the fact that a dropout analysis involving baseline determinants showed no significant differences between children lost to follow-up and those who stayed in the cohort. Furthermore, our results were based on direct and standardized length/height measurements for both children and their mothers.

## Conclusions

In conclusion, we found that socioeconomic background, a potentially modifiable factor, is a predictor of linear growth during the school-aged years, and maternal height and infant characteristics at birth influence growth throughout childhood. Because height measured during late childhood is highly correlated with adult height, interventions to enhance population health should begin by focusing on distal determinants of linear growth and consider social inequalities in early life.

## Competing interests

The authors declare that they have no competing interests.

## Authors’ contributions

BHL contributed to the study design and data collection; BHL, EV, and RAA participated in statistical data analyses; BHL conducted data analyses, interpreted results, and wrote the initial draft of the manuscript; MAC implemented and supervised all study protocols and was responsible for project management; BHL, EV and MAC participated in data interpretation and were involved in the review of the manuscript. All authors read and approved the final manuscript.

## Pre-publication history

The pre-publication history for this paper can be accessed here:

http://www.biomedcentral.com/1471-2458/12/265/prepub
